# Dynamic Drug-Induced Sleep Computed Tomography in Adults With Obstructive Sleep Apnea

**DOI:** 10.1038/srep35849

**Published:** 2016-10-20

**Authors:** Hsueh-Yu Li, Yu-Lun Lo, Chao-Jan Wang, Li-Jen Hsin, Wan-Ni Lin, Tuan-Jen Fang, Li-Ang Lee

**Affiliations:** 1 Department of Otorhinolaryngology - Head and Neck Surgery, Sleep Center, Linkou-Chang Gung Memorial Hospital, Taoyuan City 33305, Taiwan, ROC; 2 Faculty of Medicine, College of Medicine, Chang Gung University, Taoyuan City 33303, Taiwan, ROC; 3 Department of Sleep Medicine, Royal Infirmary of Edinburgh, Edinburgh EH16 4SA, UK; 4 Department of Thoracic Medicine, Sleep Center, Linkou-Chang Gung Memorial Hospital, Taoyuan City 33305, Taiwan, ROC; 5 Healthcare Center, Taoyuan-Chang Gung Memorial Hospital, Taoyuan City 33378, Taiwan, ROC; 6 Department of Medical Imaging and Intervention, Sleep Center, Linkou-Chang Gung Memorial Hospital, Taoyuan City 33305, Taiwan, ROC

## Abstract

Surgical success for obstructive sleep apnea (OSA) depends on identifying sites of obstruction in the upper airway. In this study, we investigated sites of obstruction by evaluating dynamic changes in the upper airway using drug-induced sleep computed tomography (DI-SCT) in patients with OSA. Thirty-five adult patients with OSA were prospectively enrolled. Sleep was induced with propofol under light sedation (bispectral index 70–75), and low-dose 320-detector row CT was performed for 10 seconds over a span of 2–3 respiratory cycles with supporting a continuous positive airway pressure model. Most (89%) of the patients had multi-level obstructions. Total obstruction most commonly occurred in the velum (86%), followed by the tongue (57%), oropharyngeal lateral wall (49%), and epiglottis (26%). There were two types of anterior-posterior obstruction of the soft palate, uvular (94%) and velar (6%), and three types of tongue obstruction, upper (30%), lower (37%), and upper plus lower obstruction (33%). DI-SCT is a fast and safe tool to identify simulated sleep airway obstruction in patients with OSA. It provides data on dynamic airway movement in the sagittal view which can be used to differentiate palate and tongue obstructions, and this can be helpful when planning surgery for patients with OSA.

Obstructive sleep apnea (OSA) is characterized by repeated partial or complete collapses of the upper airway during sleep^1^. Untreated OSA is associated with an increased risk of morbidity and mortality^2^,^3^. Continuous positive airway pressure (CPAP) is commonly used as the first-line therapy for OSA^4^. Surgery is currently the only treatment modality that does not require the use of a device such as CPAP or oral appliance. Surgery can enlarge airspace and decrease resistance to improve airway obstruction and related apnea, arousal, daytime sleepiness, and tense the soft tissue to decrease vibration and improve snoring. Surgical effects can also be enhanced by weight reduction, adequate sleep position, treatment of nasal allergy, and oropharyngeal muscle training. These non-surgery treatments can be tried before operation. The success of surgery for OSA depends on identifying the sites of obstruction in the upper airway, for which traditional assessment tools performed during wakefulness include physical examination, fiberoptic endoscopy with Muller’s manoeuvre, and imaging studies including cephalometry, videofluoroscopy, computed tomography (CT), and magnetic resonance imaging (MRI)^5^,^6^,^7^,^8^,^9^. However, discrepancies in the sites of obstruction in the upper airway have been reported between those detected during wakefulness and those detected during sleep^10^. Drug-induced sleep endoscopy (DISE) is increasingly being used as a preoperative examination tool for patients undergoing surgery for OSA because it can provide three-dimensional evaluations of changes in the upper airway during pharmacologically-induced sleep^11^. However, problems with DISE include the endoscope in the airway lumen interrupting the obstruction model, one site of obstruction being observed in one level of evaluation, and difficulty in precise assessment of areas of tongue obstruction.

Recent technological developments have improved the speed of CT scanning, so that fast dynamic movement of the upper airway during sedation-induced sleep can be clearly observed and scored in different sections. The purpose of this study was therefore to investigate the feasibility of detecting dynamic upper airway obstructions in patients with OSA using drug-induced sleep computed tomography (DI-SCT). The findings may be helpful in establishing an accurate assessment model for upper airway obstructions in patients with OSA to facilitate surgical planning and improve surgical outcomes.

## Results

### Study population

Thirty-five patients were enrolled in this study (34 men) with a mean age of 40 ± 10 years, a mean body mass index of 27.0 ± 3.3 kg/m^2^, and a mean apnea-hypopnea index (AHI) of 54.9 ± 28.0 events/hour. Six (17%) of the subjected had moderate OSA whereas 29 (83%) cases had severe OSA. Three (9%) patients had rapid eye movement (REM)-related OSA. Moreover, 30 (86%) patients had position-dependent OSA defined by polysomnography (PSG), and none had OSA only in the lateral position. All patients completed the DI-SCT examination with no event of airway compromise. One patient needed to repeat CT scan because arousal caused temporary termination of scanning. Thirty-one (89%) CT captured events were ‘obstructive sedation apneas’ and 4 (11%) were ‘obstructive sedation hyponea’. Images of airway obstruction in all cases were smoothly categorized according to VOTE classification^12^.

### Sites of obstruction

**Figure 1:**
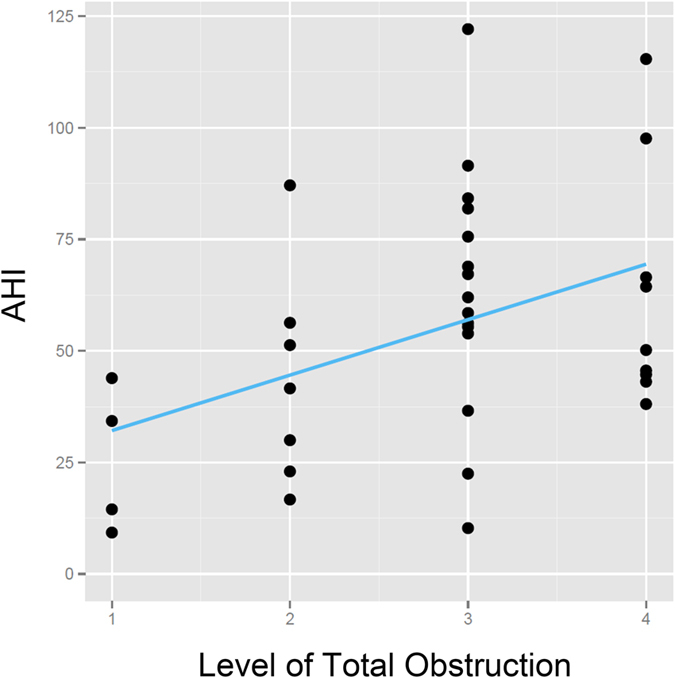
Correlation between levels of total obstruction and apnea-hypopnea index (AHI). The levels of total obstruction were significantly associated with AHI (*r* = 0.40, *p* = 0.02).

Most (89%) of the patients had multi-level total (>90%) obstructions, including 20% (7/35) with two levels, 43% (15/35) with three levels, and 26% (9/35) with four levels, and 11% (4/35) had an obstruction in one level. Among multi-level obstruction, 17 (49%) patients showed simultaneous multi-level obstruction and 14 (40%) presented sequential multi-level obstruction. The levels of total obstruction were positively correlated with AHI (*r* = 0.40, *p* = 0.02) (Fig. 1). All of the subjects had partial (50–90%) or total airway obstructions related to the velum. The most common site of total obstruction was the velum (86%, 30/35), followed by the tongue (57%, 20/35), oropharyngeal lateral wall (49%, 17/35), and epiglottis (26%, 9/35) (Table 1).

### Specific classifications of velopharynx and tongue obstructions

Anterior-posterior obstructions of the soft palate included uvular (94%, 33/35) and velar (6%, 2/35) types. Tongue obstructions (partial plus total, n = 27) included upper (30%, 8/27), lower (37%, 10/27), and upper plus lower (33%, 9/27) types.

## Discussion

This is the first study to use DI-SCT to identify dynamic obstruction of the upper airway based on VOTE classification in patients with OSA. Most of the patients had obstructions in three levels. The soft palate was the most common site of obstruction and included uvular and velar types, and the tongue was the second most common site of obstruction and included upper, lower and upper plus lower types. Other sites of obstruction included the oropharyngeal lateral wall and epiglottis in around one half and one fourth of the patients, respectively. To the best of our knowledge, OSA composes a combination of morphological and physiological changes during sleep^12^. More specifically individually surgical techniques for widening and stabilizing a small or collapsible airway can enhance treatment outcome in selected patients with OSA.

Patients with OSA generally have obstructions in more than one level, and studies using DISE have reported that multi-level obstructions occur in 76–80% of patients with moderate to severe OSA^12^,^13^. Using DI-SCT, our results showed that 89% of the patients had multi-level obstructions based on VOTE classification of the upper airway. Furthermore, most of the patients had obstructions in three levels, and the levels of obstruction were significantly correlated with AHI. These findings show that multi-level airway obstruction is common, and that the levels of obstruction are related to disease severity in patients with moderate to severe OSA. This may be because of narrowing in multiple sites of the upper airway, and/or sequential collapse due to powerful negative pressure. The involvement of multiple sites in the airway in patients with moderate to severe OSA suggests the necessity of multi-level surgery or multi-modality treatment to obtain an optimal outcome.

The velum was the most common site of airway obstruction in our OSA patients, with all having a partial or total velar obstruction and 86% having a total obstruction. These results suggest that palatal surgery is essential in the treatment of snoring (partial obstruction) and sleep apnea (total obstruction) in patients with OSA. DI-SCT provides data on dynamic airway movements in the sagittal view that can help to differentiate soft palate obstructions. Different types of palatal obstruction may need specific surgical treatment. Uvulopalatopharyngoplasty (UPPP)^14^, extended uvulopalatal flap^15^, or relocation pharyngoplasty^16^ can be helpful for the uvular type because the treatment involves the lower soft palate and uvula. For the velar type, transpalatal advancement pharyngoplasty^17^ or modified cautery-assisted palatal stiffening surgery^18^ plus UPPP is suggested to advance the whole soft palate.

The tongue was the second most common site of obstruction, accounting for more than 50% of obstructions in our patients. Previous studies have described tongue obstructions as retroglossal (or hypopharyngeal) and focused on the tongue base. In the current study, three types of tongue obstruction (upper, lower, and upper plus lower) were identified. Previous studies on DI-SCT have reported that 30% of patients with tongue obstructions present with upper tongue obstructions in which the tongue body pushes the soft palate backward during sleep, and that this is commonly recorded as an anterior-posterior palatal obstruction during DISE^13^,^19^. Tongue base reduction surgery may not be helpful in these patients due to worsening of the tongue collapse. Lower tongue obstructions occurred in only 37% of patient with tongue obstructions, suggesting that hypopharyngeal obstructions are not limited to merely the tongue base level. Tongue base reduction or suspension surgery has been reported to be helpful in these patients^20^,^21^. Furthermore, 33% of the patients with tongue obstructions had complex obstructions in both the upper and lower tongue. This suggests that comprehensive tongue surgery involving both the body and base of the tongue is warranted in these patients. In our previous studies^20^,^21^,^22^, we presented our surgical techniques for tongue reduction surgery involving the tongue body and base, and reported that outcomes of tongue reduction surgery involving the tongue body and base was superior to tongue base surgery (lingual tonsillectomy) in adults with OSA^20^. The results of DI-SCT in this study show that it can help to identify the type of tongue obstruction which may otherwise be difficult in intraluminal examinations with DISE.

Obstruction of the lateral pharyngeal wall (LPW) occurred in nearly half of our patients, which is consistent to reports on DISE^23^. Collapses in the LPW of the hypopharynx are difficult to approach using traditional UPPP, which limits its use for patients with OSA. Relocation pharyngoplasty^16^ or lateral pharyngoplasty^24^ with stabilization of the LPW in the inferior tonsillar fossa may be partially effective in decreasing LPW obstructions. However, maxillomandibular advancement is the preferred form of treatment by tensing the LPW^25^.

Epiglottis collapse during sleep has been estimated to occur in around 12% of adult patients with OSA^26^. However, with the use of DISE, anterior-posterior epiglottis collapse has been found to occur more frequently than in previous reports^27^. In the present study, 25% of the patients had collapse of the epiglottis, which is consistent with the studies using DISE, and this highlights the need for drug-induced sleep airway examinations to identify collapse of the epiglottis. Positional therapy seems to offer good results^28^, and epiglottidectomy has been reported to provide the most effective solution to treat epiglottis collapse^29^. Further, the high occurrence rate of epiglottis collapse in patients with OSA can partially explain the residual AHI in these patients after multi-level surgery, which usually does not include procedures for the epiglottis^30^.

There are four major limitations to this study. First, drug-induced sleep may not represent natural sleep. A previous study reported that upper airway obstruction during drug-induced sleep is dose related, and that a higher dose causes increased severity of the collapse and levels of the upper airway^31^. In this study, the depth of sedation was monitored using a bispectral index system, and we chose 70–75 (light sedation) to avoid unnecessary collapse of the upper airway due to drug-induced reductions in muscle tone^32^. Moreover, propofol possibly induced non-REM sleep and might have affected the sites of obstruction observed and the surgical procedure planned in OSA patients with only REM-related events. Second, concerns of tissue damage from high doses of radiation for CT scans may limit its wide use. We used dynamic CT scans with a relatively low radiation dose (1.37 mSv) which is lower than the mean effective dose from regular head CT scan (2 mSv) and considered to be tolerable and safe for patients^33^. Sleep MRI is another option which does not require ionizing radiation^34^. However, the loud noises during MRI scanning may interrupt drug-induced sleep. In addition, MRI is time-consuming and is thus likely to increase the risk of airway compromise during sedation in patients with OSA. In addition, vital signs and BIS monitoring is needed to ensure patient safety. Furthermore, dynamic MRI is not three-dimensional at one time. Accordingly, we chose CT despite the exposure to radiation. Third, short-term upper airway images may not present the whole profile of airway obstructions in different stages of sleep. It is genuine that airway lumen is different in individual sleep stage due to the change in muscle tone. Light sedation used in this study may not reflect the whole profile in the changes of muscle tone and collapsibility during sleep. Further study to include extensive drug-induced sedation for CT scan may observe more comprehensive airway changes during simulated sleep. Forth, the cohort of this study cannot reflect on all OSA patients, but instead on overweight primarily men that could not tolerate CPAP or were unwilling to try it.

In summary, DI-SCT is a fast and safe examination tool to identify airway obstructions during simulated sleep in adult patients with OSA. DI-SCT can provide data on dynamic airway movement in the sagittal view which can then facilitate differentiating palate and tongue obstructions, which may be helpful when planning surgery to improve treatment outcomes.

## Methods

### Ethics statement

The Institutional Review Board of Linkou-Chang Gung Memorial Hospital, Taoyuan, Taiwan approved this study (101–3547A3). All procedures were carried out in accordance with the current regulations. All of the subjects provided written informed consent to participate in this study.

### Study population

This prospective study recruited 35 patients with OSA between 2012 October and 2014 September. The inclusion criteria were: (1) chief complaint of snoring and/or daytime sleepiness; (2) moderate to severe OSA (AHI >15); and (3) age between 18–60 years. The exclusion criteria were: (1) allergy to propofol or history of seizure; (2) pregnant or lactating women; (3) poor general condition for surgery such as stroke, coronary heart disease, bleeding tendency, chronic obstructive pulmonary disease, uncontrolled asthma, neuromuscular disease, or pathological obesity; (4) altered upper airway anatomy; and (5) working abroad or incapable of regular follow-up. All of the participants were either intolerant or unwilling to undergo long-term CPAP therapy and were referred for surgical evaluation. In our sleep center, we always recommended sleep apnea patients with central AHI ≥ 5 events/h or central/mixed events ≥25% of total events to undergo CPAP therapy instead of upper airway surgery. The surgical plan for each patient was based on their anatomical structure and disease severity, and was fully discussed with them before making the decision to undergo surgery.

### PSG

Level I PSG (Nicolet UltraSom System, Madison, Wisconsin, USA) was performed in a laboratory to document sleep and breathing in all patients. PSG included electroencephalogram, electro-oculography, nasal and oral airflow measurements, assessment of thoracic and abdominal movements, and evaluation of oxyhemoglobin saturation. The main PSG parameter used in this study was AHI. Apnea was defined as a drop in the peak thermal sensor excursion by at least 90% of baseline for at least 10 seconds. Hypopnea was defined as a decrease in the nasal pressure signal excursion by ≥30% for at least 10 seconds accompanied by desaturation of 4% or more from pre-event baseline or an arousal from sleep^35^.

### CPAP titration

**Figure 2:**
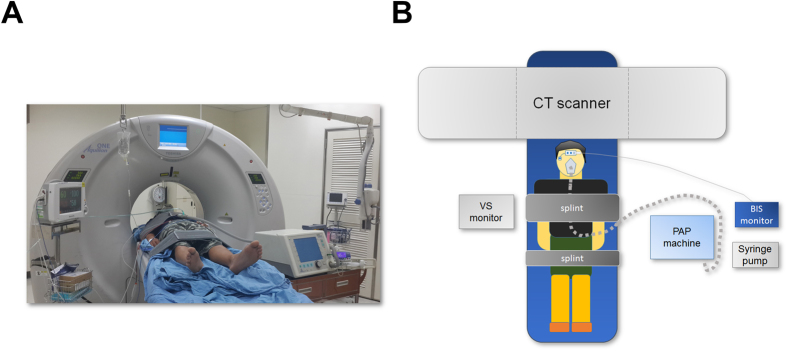
Setting of drug-induced sleep computed tomography (DI-SCT). (**A**) DI-SCT facility. (**B**) A diagram showing the relative position of monitors for vital signs, sedation depth, and continuous positive airway pressure during DI-SCT.

The procedure used for drug-induced sleep in a CT room unit equipped with standard anaesthetic monitoring (oxygen saturation, non-invasive blood pressure, and electrocardiography) was similar to our previous reports^36^,^37^. Despite drug-induced sleep has been proven its safety in severe OSA patients^38^, we closely monitored the occurrence of mean arterial pressure <65 mmHg, oxygen saturation <90%, and persistent apnea. We could provide immediate interventions such as positional therapy, fluid challenge, epinephrine, oxygen supplement, CPAP therapy, and resuscitation when indicated. Risk and management have been illustrated in the informed consent of this study in accordance with the current regulations. In addition, written ‘Informed Consent for Moderate/Deep Sedation’ have been obtained from all participants before the procedure of DI-SCT. Patients with a full face mask connected to a positive airway pressure (PAP) machine were placed in the supine position with their head in a natural position in the CT room (Fig. 2A). The depth of sedation was monitored by an A-2000 bispectral index (BIS)-Vista monitor (Version 3.11, Aspect Medical Systems, Inc., Newton, MA). All patients received propofol (10 mg/mL, AstraZeneca, Caponago, Milano, Italy) sedation by the same doctor (Y.L. LO). Intravenous propofol was administered with the initial administration of 0.5 mg/kg via a syringe pump (Injectomat Agilia, Fresenius Kabi, France) and further doses of 10–20 mg were given every 30 seconds to achieve the target level of sedation (BIS value: 70–75) for CT scanning^36^. CPAP with the lowest pressure of 4 cmH_2_O was given to every patient while awake as an accommodative pressure before DI-SCT. The pressure of the CPAP model was gradually increased to maintain airway patency when the depth of sedation deepened. It was then reduced back to 4 cmH_2_O after the patient reached the target sedation depth for DI-SCT scanning. For the prevention of propofol-induced central events due to over-ventilation or arousal, we maintained stable ventilation and steady sedation level for one minute before we dialed down the pressure to baseline. Vital signs, sedation depth, and CPAP pressure were monitored during the whole DI-SCT procedure (Fig. 2B). In the DI-SCT scenario, the researchers saw patients had increasing inspiratory efforts accompanied with (1) a drop in the peak signal excursion by ≥90% of pre-event baseline using PAP flow (obstructive apnea) or (2) several flow-limited breaths (drops in the peak signal excursions by ≥30% of pre-event baseline) with a ‘peak-plateau’ inspiratory flow pattern and ≥4% oxygen desaturation (obstructive hypopnea) from a liquid-crystal display of the PAP machine then leaved the CT room and started the CT scan for images required. By this process, images spanning partial and total obstruction of the airway were adequately captured. After the scan, the pressure was increased back to the previous highest pressure (range 12–20 cmH2O) before dialling down to prevent airway collapse until the patient awoke. The patients were then moved to an observation room where their vital signs (pulse oximeter, blood pressure and electrocardiography) were measure, before they were finally discharged in a fully awake status with normal respiration.

### Dynamic CT scan

**Figure 3:**
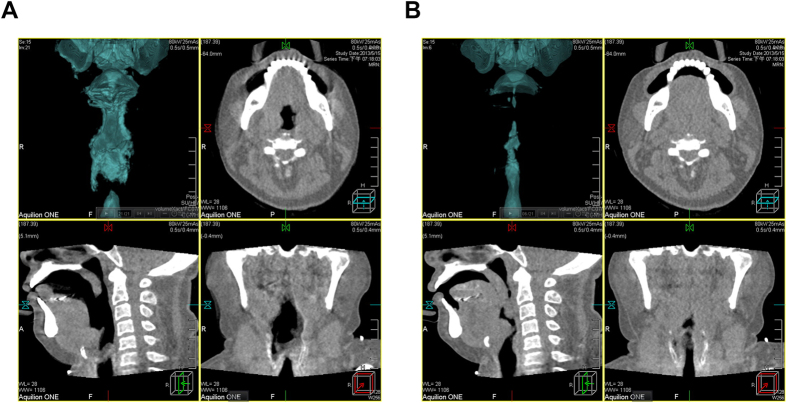
A representative case of severe obstructive sleep apnea. Raw data were reconstructed and transferred to a workstation for post-processing, including mid-sagittal and three-dimensional, and dynamic display in wakefulness (**A**) and drug-induced sleep (**B**).

CT images were obtained using an Aquilion One system (320-detector row, Toshiba, Japan) using a dynamic volume scan protocol when the patients were awake and in drug-induced sleep. DI-SCT was performed when light sedation (BIS 70–75) with propofol had been accomplished. The dynamic scan from the orbital floor to the hyoid bone continued for 10 seconds and spanned 2–3 respiration cycles. The time period of 10 seconds for DI-SCT was chosen with the concern for both radiation dose and respiration cycles. The radiation dose was 1.37 mSv. Raw data were reconstructed and transferred to a workstation for post-processing, including mid-sagittal and three-dimensional, dynamic display as well as virtual endoscopy (Fig. 3).

### Main outcome measurements

The primary outcome was the site of the upper airway obstruction during DI-SCT. The dynamic collapse of the upper airway in DI-SCT was assessed in four sites: the velopharynx, oropharyngeal lateral wall, tongue, and epiglottis based on the VOTE classification used in drug-induced sleep endoscopy^12^. The collapse score was defined as: 0 (<50% collapse of the airway, none); 1 (50–90% collapse of the airway, partial); and 2 (>90% collapse of the airway, total). A single observer who was blind to the polysomnographic results graded the collapse scoring of this series with the aforementioned criteria for consistency.

**Figure 4:**
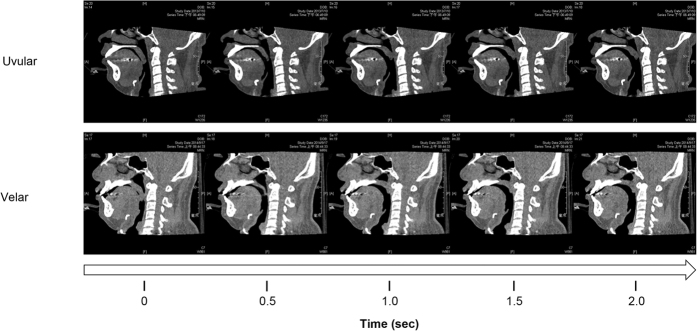
Types of anterior-posterior palatal obstruction. Two types (uvular and velar) of anterior-posterior palatal obstruction were classified in drug-induced sleep computed tomography.

**Figure 5:**
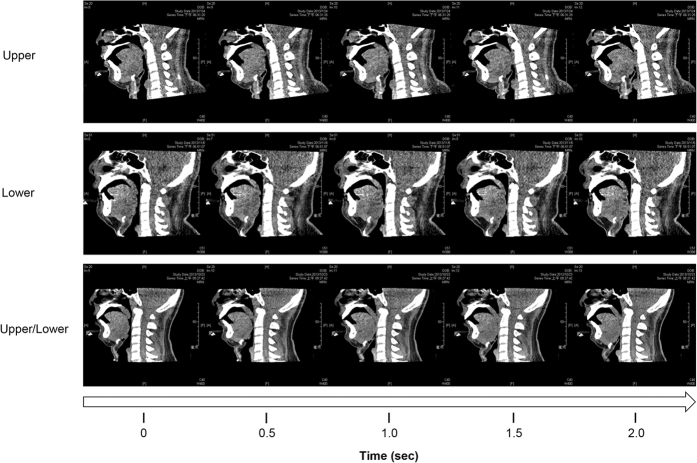
Types of tongue obstruction. Upper, lower and upper/lower types of tongue obstruction were noted in drug-induced sleep computed tomography.

The secondary outcome measurements included the ‘total DI-SCT score’ and the patterns of the anterior-posterior obstruction of the soft palate and tongue. The total DI-SCT score was calculated by the sum of levels of total obstruction. In addition, the patterns of the anterior-posterior obstructions during dynamic observations were further subdivided into ‘uvular type’ (lower part of the soft palate and uvula moving backwards to contact the posterior pharyngeal wall) and ‘velar type’ (lower soft palate obstruction occurring first, followed by upper soft palate obstruction) for the soft palate (Fig. 4), and ‘upper obstruction’ (tongue body moving backwards to compress the soft palate and obstruct the airway), lower obstruction (tongue base/lingual tonsils moving backwards to contact the posterior pharyngeal wall and obstruct the airway), and upper plus lower obstruction (upper tongue obstruction occurring first, followed by lower tongue obstruction) for the tongue (Fig. 5).

### Statistical analysis

Normally distributed data were reported as a mean ± standard deviation (SD). The degree of correlation between levels of total obstruction and AHI was assessed using the Spearman correlation test. Statistical analyses were performed using IBM SPSS software (version 23; International Business Machines Corp., Armonk, NY, USA). All *p* values were two-sided, and statistical significance was accepted at *p* < 0.05.

## Additional Information

**How to cite this article**: Li, H.-Y. *et al*. Dynamic Drug-Induced Sleep Computed Tomography in Adults With Obstructive Sleep Apnea. *Sci. Rep.* **6**, 35849; doi: 10.1038/srep35849 (2016).

## Figures and Tables

**Table 1:** Upper airway collapse during drug-induced sleep computed tomography.

VOTE^19^ (*n* = 35)	0 (<50%)	1 (50–90%)	2 (>90%)
Velopharyngeal obstruction	0 (0%)	5 (14%)	30 (86%)
Oropharyngeal LW obstruction	8 (23%)	10 (29%)	17 (49%)
Tongue obstruction	8 (23%)	7 (20%)	20 (57%)
Epiglottis obstruction	20 (57%)	6 (17%)	9 (26%)

Note: Numbers are n (%).

VOTE, velopharynx, oropharyngeal lateral wall, tongue and epiglottis; LW, lateral wall.
